# Soil Organic Matter Estimation Model Integrating Spectral and Profile Features

**DOI:** 10.3390/s23249868

**Published:** 2023-12-16

**Authors:** Shaofang He, Siqiao Tan, Luming Shen, Qing Zhou

**Affiliations:** 1 College of Information and Intelligence, Hunan Agricultural University, Changsha 410128, China; 2 College of Resources, Hunan Agricultural University, Changsha 410128, China

**Keywords:** fusion feature, PCA, lasso feature selection, SCARS

## Abstract

The accurate measurement of soil organic matter (SOM) is vital for maintaining soil quality. We present an innovative model for SOM prediction by integrating spectral and profile features. We use PCA, Lasso, and SCARS methods to extract important spectral features and combine them with profile data. This hybrid approach significantly improves SOM prediction across various models, including Random Forest, ExtraTrees, and XGBoost, boosting the coefficient of determination (R^2^) by up to 26%. Notably, the ExtraTrees model, enriched with PCA-extracted features, achieves the highest accuracy with an R^2^ of 0.931 and an RMSE of 0.068. Compared with single-feature models, this approach improves the R^2^ by 17% and 26% for PCA features of full-band spectra and profile features, respectively. Our findings highlight the potential of feature integration, especially the ExtraTrees model with PCA-extracted features and profile features, as a stable and accurate tool for SOM prediction in extensive study areas.

## 1. Introduction

Soil organic matter (SOM) content serves as a pivotal metric for gauging soil fertility and evaluating the quality of arable land, as well as for comprehending soil carbon cycles and managing soil degradation [1]. The prevailing laboratory technique for determining SOM content is the potassium dichromate volumetric method—a widely accepted standard approach noted for its expeditiousness, simplicity, and accuracy in yielding minimal experimental errors [2]. However, this method is marred by an intricate operational process and the generation of hazardous byproducts that can impinge on human health. Presently, spectral remote sensing stands as a prominent tool for estimating soil trait indicators. Indoor visible-near-infrared spectra of soil samples, under controlled environmental conditions, offer attributes such as high-resolution multiple and continuous bands, rendering them potent for estimating traits like organic matter content. Consequently, the quest for swiftly acquiring SOM content using spectral analysis has garnered scholarly attention. Notably, when using full-band soil spectra for SOM content-estimation models, it has been observed that not all spectral data bands exhibit robust responses to SOM content [1]. Discarding redundant or unreliable bands and capturing sensitive spectral features or regions not only diminishes computational complexity but also furnishes a SOM estimation model with superior fitting capabilities. Standard practice demands spectral data preprocessing prior to quantitative SOM content analysis. This includes procedures like the logarithmic transformation of spectral reflectance for noise amplification [3], noise mitigation using the Savitzky–Golay smoothing algorithm [4], nullifying the influence of soil sample particle size using the standard orthogonal transform [5], and using wavelet transforms for spectral calibration [3]. The predominant methods for selecting sensitive spectral bands in soil spectra entail correlation analysis [6], Principal Component Analysis (PCA) [7], Stability Competitive Adaptive Reweighted Sampling (SCARS) [8], the Successive Projection Algorithm (SPA) [9], Uninformative Variable Elimination (UVE), and the Genetic Algorithm (GA) [10]. Traditionally, these feature variable screening methods are applied independently in spectral data analysis. However, combining them can lead to unexpected outcomes. For instance, the concurrent use of competitive adaptive reweighted sampling (CARS) with SPA resulted in the coefficient of determination (R^2^) reaching as high as 0.92 on a prediction set [11]. Moreover, the intricacies of soil organic matter content extend due to varying soil profile elements’ soil-forming parent material, land type, and elevation. Hence, beyond spectral analysis, select scholars have delved into the interplay between diverse soil profile attributes and organic matter content across distinct ecosystems [12,13,14,15]. For instance, in reference [16], the researchers constructed a multivariate stepwise linear regression and back propagation neural network prediction model for soil organic matter content. They utilized PCA to reduce the measured hyperspectral data into six principal components and integrated extracted moisture and vegetation spectral feature indices as independent variables. This integration resulted in an uplift effect of up to 6.1%. This exploration augments the prediction of spatial soil organic matter distribution.

Regression techniques used to estimate SOM content based on soil spectral data and diverse levels of soil profile attributes encompass both linear and nonlinear modeling approaches [17,18]. Notably, linear methodologies frequently embraced include partial least squares regression (PLSR) and linear regression (LR) [19]. Moreover, select investigations have extended to encompass regression models like Principal Component Analysis and Decision Trees [20]. In parallel, nonlinear models play a pivotal role and encompass Support Vector Machine (SVM) [21], Random Forest (RF) [22], and Artificial Neural Network (ANN) models [23]. These sophisticated methods capitalize on the intricate relationships present within the data, allowing for more intricate and nuanced predictions.

Broadly, substantial progress has been made in estimating SOM content using spectral analysis techniques and soil profile data. Nevertheless, challenges persist when translating quantitatively inverted indoor spectral organic matter models into field environmental conditions. This stems from intricate control over diverse environmental factors and neglecting the impact of soil environmental elements on organic matter content. Consequently, these models are not ideally suited for extensive-scale SOM monitoring. Comparatively, SOM content estimation models rooted in diverse profile characteristics prove to be more applicable to large-scale regional studies. However, these models primarily focus on field environmental influences on organic matter content, disregarding spectral variations prompted by soil sample physiological responses to the environment. Consequently, the efficacy of these models for SOM inversion is limited. To address the conundrum posed by single-feature soil sample-based SOM inversion models (indoor spectral or multi-level profile features), this study endeavors to ameliorate predictive performance by integrating disparate types of features. This pursuit involves the amalgamation of spectral features with profile features, offering a more comprehensive and nuanced understanding of SOM dynamics. The ensuing framework aims to enhance prediction accuracy and applicability within large-scale regional contexts. This investigation embraces a multifaceted methodology involving spectral analysis, soil profile information, and various regression algorithms. The study area encompasses the rice-growing soils of paddy fields in Changsha and Zhuzhou, Hunan Province, characterized by diverse soil types, elevation, and pH values. Soil samples collected from this region were subjected to meticulous data preprocessing, feature selection, and model construction. Three distinct feature extraction methods were used to curate meaningful subsets of features from the full-band spectra: PCA, the Lasso method, and the SCARS technique. The resultant feature sets were then integrated with soil profile information to create fused features, which were subsequently used in concert with regression algorithms to construct SOM content prediction models.

The subsequent content is structured as follows: Section 2 provides an exposition of the data sources, methods, and models used. The evaluation and comparative analysis of these models on the dataset are presented in Section 3. Section 4 offers a comprehensive discussion of stability for Lasso-selected features modeling and SCARS-PCA features modeling. Finally, Section 5 offers concluding remarks for this comprehensive paper.

## 2. Materials and Methods

### 2.1. Soil Profile Data and Spectral Data Acquisition

The focal study area in this research encompasses the rice-cultivating soils within and surrounding Changsha and Zhuzhou, located in Hunan Province (112.608–114.067 E, 27.536–28.514 N). The soil texture is predominantly clay-based. The topography of the study area is predominantly flat, spanning an elevation range of 30.75 to 164 m. The soils exhibit good drainage characteristics, with pH values ranging from 4.5 to 9.0. In the context of soil profile descriptions, “CS” denotes Changsha, while “CS-03” designates the third sampling site within the Changsha area. Further elaborating, “CS-03-Aa”, “CS-03-Ap”, “CS-03-B”, “CS-03-Br”, “CS-03-Bg”, and “CS-03-Er” serve as distinct labels for each occurrence layer within the CS-03 sampling site. Each soil profile at a given sampling site is segmented into five to seven layers based on depth, and these layers are identified using their respective occurrence layer notations. Taking the example of CS-03, Table 1 displays the specifics of the different profile levels. Each soil sample comprises 12 distinct attribute features. The primary attribute of CS-03, Profile_level, categorizes the sampling depth into six classes, denoted by values 1 through 6, signifying depths of 0–10 cm, 11–20 cm, 21–30 cm, 31–50 cm, 51–70 cm, and 71–90 cm, respectively. Color_class, Color_value, and Color_chroma delineate color attribute characteristics of soil samples under wet conditions, representing hue class, color value, and chroma, respectively. Soil colors are systematically described using the Munsell chart with three key parameters: hue (representing the actual color), value (indicating the lightness or darkness), and chroma (illustrating the color intensity). Each hue is quantified on a scale of 2.5, 5, 7.5, and 10. In our soil sample, the identified hue is yellow-red (YR). The values delineate the luminance range from absolute black (0, no light reflection) to absolute white (10, reflecting all light). Chroma measures a color’s tonal strength concerning a neutral grey at an identical luminance level. The attributes Plant_root_thickness and Plant_root_abundance pertain to the characteristics of the plant root system, reflecting the thickness and abundance of roots, respectively. Degree_of_soil_structure_development quantifies the extent of soil structure development, offering insights into its progression. Porosity, Pore_size, and Pore_abundance describe attributes associated with soil pore space, encapsulating porosity, pore thickness, and abundance, respectively. Plasticity measures a soil sample’s plasticity in its wet state, while pH signifies the soil’s acid-base value.

A total of 79 soil samples were systematically collected from rice fields within the designated study area, using a handheld Global Positioning System (GPS) locator for precise positioning. Subsequently, in the laboratory setting, these samples were air-dried and finely milled. Each soil sample underwent partitioning into two segments—one earmarked for spectral data collection and the other reserved for physicochemical property analysis. To ascertain the organic matter content within the soil samples, the potassium dichromate external heating method was used. Notably, each sample underwent this determination procedure twice, and the resultant values were averaged. Statistical analysis of the data revealed that the organic matter content spanned a substantial range. Specifically, the organic matter content exhibited a minimum value of 2.6 g/Kg and a maximum value of 45.5 g/Kg.

**Figure 1:**
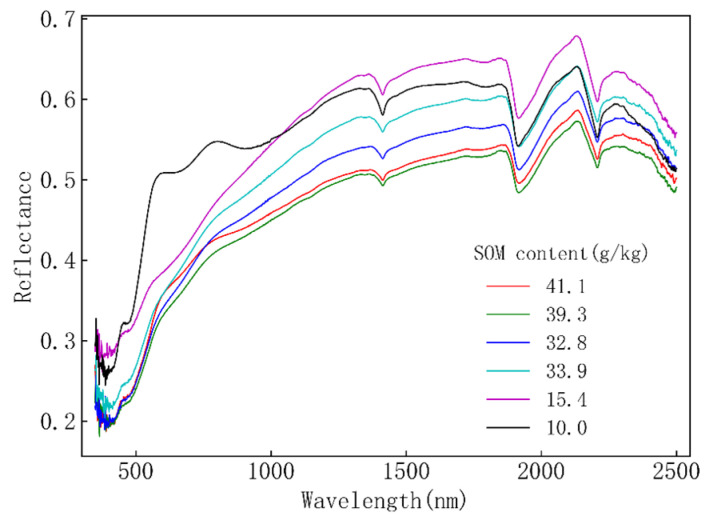
Spectral reflectance of different SOM contents in CS03.

Spectral data were collected using an ASD Vis-NIR spectrometer, boasting a wavelength range spanning from 350 to 2500 nm. Notably, the sampling intervals were set at 1.4 nm within the 350–1000 nm range and 2.0 nm within the 1000–2500 nm range, complemented by resampling intervals of 1 nm. The indoor measurement of spectral data from all 79 soil samples was executed and further subjected to noise reduction with the application of the Savitzky–Golay smoothing algorithm, effectively mitigating noise artifacts. Spectral reflectance of the soil profiles exhibited dynamic variability across occurrence layers, reflecting differences in organic matter content. Illustratively, within the CS03 profile, the organic matter content (measured in g/Kg) across the six occurrence layers—CS-03-Aa, CS-03-Ap, CS-03-B, CS-03-Br, CS-03-Bg, and CS-03-Er—stood at 41.1, 39.3, 32.8, 33.9, 15.4, and 10, respectively. These distinct organic matter contents are visually represented in Figure 1, highlighting how spectral reflectance patterns vary based on organic matter content. Evidently, spectral reflectance curves manifest notable resemblances across different soil organic matter contents.

### 2.2. Predictive Modeling of Soil Organic Matter Content Using Fusion Features

**Figure 2:**
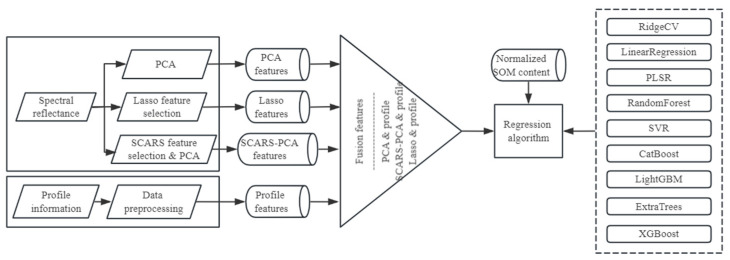
Model framework.

The schematic representation of the integrated soil SOM content prediction model, incorporating both spectral and profile features, is depicted in Figure 2. Predicting SOM content entails a regression challenge, and the model’s conception is as follows.

1. Data preprocessing: The diverse levels of soil profile information from sampling points within the study region are collated, accompanied by the laboratory-measured organic matter content of soil samples and spectral data. The leave-one-out method is used, coupled with normalization techniques, to preprocess the profile information, thereby deriving distinctive profile features.
2. Feature extraction: The risk of overfitting escalates when the number of features surpasses the quantity of samples. To circumvent this issue, we integrate the PCA technique into our feature extraction methodology. This involves utilizing PCA to downscale both the full-band spectra and the feature bands selected with SCARS. PCA is used to extract the principal component features, and the Lasso method and SCARS feature selection technique are used to extract pertinent features from the comprehensive full-band spectra. These principal component features and selected bands are subsequently merged with the profile features, yielding three sets of combination features—namely, PCA features-fused profile features, Lasso features-fused profile features, and SCARS-PCA features-fused profile features.
3. Model construction: The combination features are integrated with a regression algorithm to construct the SOM content prediction model based on fused features and normalized soil organic matter content.

#### 2.2.1. Data Preprocessing

The sampling point profiles encompass twelve features, the majority of which are categorical in nature. To encode these features, this study uses the leave-one-out method. However, some encoded profile features exhibit significant standard deviations, potentially leading to the presence of singular sample data. Such instances could prolong model training times or even impede convergence. To mitigate this, the normalization technique Min–Max Scaling is used. This process scales the data, coercing them to conform to the [0, 1] range and adhere to a normal distribution. For instance, considering sampling point CS-03 (profile information available in Table 1), the outcomes obtained after data preprocessing are detailed in Table 2. It is essential to highlight that the feature “Profile_level”, signifying the occurrence layer within the profile, is preprocessed in such a way that lower raw values yield larger coded outcomes. Notably, a correlation emerges: smaller sampling depths (aligning with smaller profile-occurring layers) correspond to larger SOM content values. As a result, the transformation of “Profile_level” into a positive feature is requisite.

#### 2.2.2. Feature Variable Selection Method

The extensive soil hyperspectral data, characterized by high-band dimensionality, contains invalid, redundant, and overlapping spectral information. This complexity poses challenges, causing instability and hindering efforts to enhance the accuracy of soil organic matter content inversion models constructed solely on the full-band basis. Constituting a pivotal facet of spectral analysis, the judicious selection of features that exhibit robust responses to soil organic matter content from the realm of redundant and high-dimensional wavelength variables directly governs the efficacy of the prediction model. In the practical course of feature extraction, the rationale is typically evaluated through two primary lenses, namely, interpretability of the target variable and redundancy among independent variables. The interpretability of the target variable emphasizes the predictive capability of individual variables or their amalgamation. Attention is paid to how well the chosen variables facilitate an understanding of the target variable. Focusing on striking a balance between model performance and diminished variable redundancy is key. This aspect seeks to ensure a streamlined and effective model outcome. Considering the inherent advantages of PCA, Lasso, and SCARS feature selection methods—specifically, their speed and the ease of interpreting selected feature variables—this study utilizes these three methodologies to navigate the extensive full-band spectra. The use of these methods stands to enhance the model’s robustness and precision.

- PCA feature extraction

PCA stands as a widely used technique for reducing the dimensionality of sample data, finding extensive application in data analysis and machine learning domains. The fundamental aim of PCA lies in substituting numerous variables in the initial dataset with a reduced set, effectively diminishing feature dimensions while conserving the bulk of sample data information. Using PCA for data dimensionality reduction not only simplifies complexities but also streamlines computer processing by reducing data volume, thereby trimming down processing times. At its core, PCA operates by maximizing the variance in sample point projections along the axes (variables) of a newly defined coordinate system achieved with axis rotation. The first principal components correspond to the coordinates of sample points along the axes with the highest variance.

For a dataset $X_{n\times p}$ encompassing *p* variables and *n* samples, the computation involves deriving the covariance matrix $\sum_{p\times p}$. Subsequently, *p* eigenvalues, denoted as $\lambda_{1},\lambda_{2},\ldots ,\lambda_{p},$ and their corresponding eigenvectors $T_{1},T_{2},\ldots ,T_{p}$ are extracted. The *k*th principal component $Y_{k}$ can be expressed as $Y_{k}=X.T_{k},$ where 1 ≤ k ≤ p.

Determining the number of selected principal components hinges upon their contribution rates and cumulative contribution. The contribution rate of the *k*th principal component, denoted as $\varphi_{k}$, is calculated as $\varphi_{k}=\frac{\lambda_{k}}{\sum_{i=1}^{p}\lambda_{i}}$. Typically, a higher contribution rate signifies a greater preservation of the original sample information. The cumulative contribution rate of the first *m* principal components $\Psi_{m}$ is computed as $\Psi_{m}=\sum_{k=1}^{m}\varphi_{k}$. A cumulative contribution rate of 80% or more suggests strong retention of the original sample information by the selected principal components, serving as a criterion for component selection.

In our indoor spectral data collection for soil samples, comprising 2151 bands (variables), we used the PCA method to select five principal components. Their respective contribution rates were 86.99%, 5.91%, 4.88%, 1.21%, and 0.49%. The resulting cumulative contribution rate stands impressively high at 99.48%, indicating an excellent retention of the original sample information.

- Lasso feature selection

Feature selection, a process entailing feature reduction, is effectively attained by introducing a penalty term, also referred to as regularization, into the loss function. This augmentation integrates the magnitude of regression coefficients into the training and parameter-solving process. The penalty coefficients are strategically set to nullify the impact of regression coefficients with limited significance, effectively attenuating them to zero. This selective approach ensures the retention of only pivotal features. The objective function of Lasso regression is succinctly depicted by Equation (1).

(1) $${min}_{\beta_{0},\beta }\{\frac{1}{2n}\sum_{i=1}^{n}{\left(y_{i}-\beta_{0}-x_{i}^{T}\beta \right)}^{2}+\alpha \sum_{i=1}^{p}\left|\beta_{j}\right|\}$$

where *n* represents the number of samples, *p* signifies the number of features, *y_i_* denotes the target variable for the *i*th sample, *β*_0_ and *β* symbolize regression coefficients, *x_i_* signifies the feature vector for the *i*th sample, and α stands for the regularization parameter.

**Figure 3:**
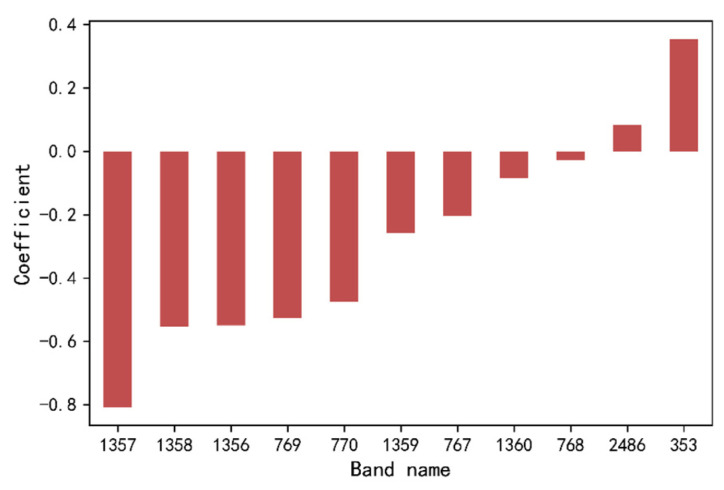
Spectral feature wavelengths selected with Lasso.

In the pragmatic execution of Lasso feature variable selection, a higher penalty coefficient results in the identification of fewer features. In this study, the cross-validation technique is harnessed to compute the Root Mean Square Error (RMSE) of the model. The optimal penalty parameter value is ascertained by identifying the juncture where the RMSE achieves an exceedingly minuscule value. The cross-validation process entails a penalty parameter selection range, specifically α = [0.001, 0.002, 0.005, 0.01, 0.1, 1.0]. Using the Lasso feature selection method, a total of eleven feature wavelengths were discerned from the comprehensive full-band spectra. Notably, the optimal penalty parameter in this context was found to be α = 0.001. This selection results in approximately 0.5% of the total wavelengths being retained. The precise positions of these wavelengths, along with their corresponding regression coefficients, are visually presented in Figure 3.

- SCARS feature selection

SCARS, a method that amalgamates Monte Carlo sampling with the PLSR model, hinges upon variable stability as the bedrock of its feature selection process. Leveraging a sequence of competitive adaptive reweighted sampling iterations, SCARS pursues variable retention criteria based on the absolute weights of regression coefficients within the PLSR model. The procedure advances iteratively by generating new subsets with adaptive reweighted sampling (ARS) while prioritizing variables with more substantial absolute regression coefficient weights. Each iteration entails the reconstruction of the PLSR model using the updated subset, culminating in the identification of wavelength variables within the subset exhibiting the smallest RMSE. The successive calculations lead to the discernment of characteristic wavelengths. In summary, the SCARS methodology operates through the following sequential steps.

Step 1: Use the Monte Carlo sampling technique to compute the stability of the *i*th wavelength variable in the *M*th Monte Carlo sample, denoted as *C_i_*. This stability measure is defined as follows:

(2) $$C_{i}=\left|\frac{{\bar{b}}_{i}}{s\left(b_{i}\right)}\right|,i=1,2,\cdots ,P$$

In this equation, ${\bar{b}}_{i}$ signifies its mean value across all Monte Carlo samples, $s\left(b_{i}\right)$ denotes the standard deviation of the *i*th wavelength variable, and *P* represents the number of variables. Evidently, higher values of ${\bar{b}}_{i}$ and lower values of $s\left(b_{i}\right)$ contribute to greater stability of the *i*th wavelength variable.

Step 2: Use forced wavelength selection and ARS to distill a subset of wavelength variables characterized by enhanced stability. Concurrently, leverage the Exponentially Decreasing Function (EDF) to quantify the ratio of retained wavelength variables relative to the entirety of the wavelength.

During each sampling iteration, the ARS method was utilized to iteratively sift wavelength variables from the subset retained in previous iterations (i.e., from steps 1 and 2). This iterative process was looped to yield a subset consisting of *K* wavelength variables (*K* representing the number of loops). Drawing upon these variable subsets, distinct PLSR models were constructed. Subsequently, the corresponding Root Mean Square Error of Cross-Validation (RMSECV) was computed. Ultimately, the wavelength variable subset that yielded the minimum RMSECV emerged as the final curated feature variable.

**Figure 4:**
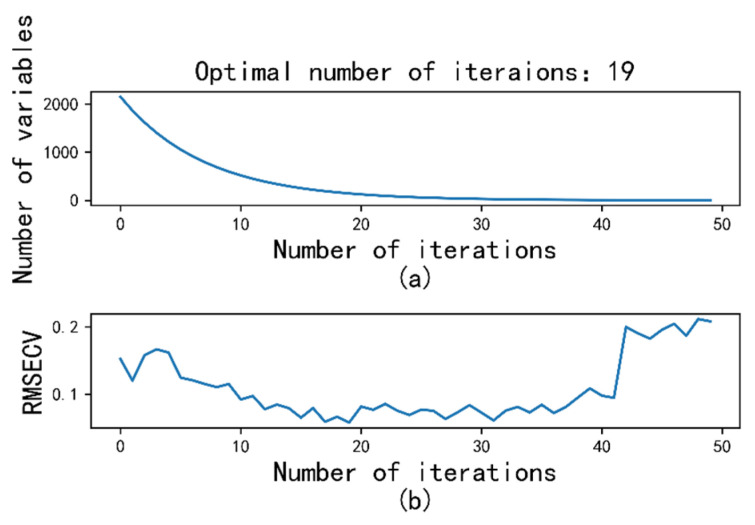
Variable selection process using SCARS. (**a**) Changing trend in the variables; (**b**) 10-fold RMSECV.

The SCARS methodology was adeptly used to distill 94 distinctive wavelengths from the expansive full-band spectrum, constituting approximately 4% of the total wavelength count. This meticulous wavelength selection process is vividly illustrated in Figure 4. As depicted in Figure 4a, the count of retained wavelengths displays a decremental trend as the number of SCARS iterations escalates. The rate of reduction transitions from swift to gradual. Meanwhile, Figure 4b depicts the trajectory of the 10-fold RMSECV in relation to the augmentation of iteration numbers. This trajectory is characterized by a sequence of shifts from higher to lower values, punctuated by minor oscillations, followed by a subsequent shift back to higher values. This pattern emerges as a progressive sequence of descending values, coupled with intermittent fluctuations, and subsequently transitioning to ascending values. Notably, a minimum RMSECV value is achieved after 19 iterations, effectively designating the resultant wavelength subset as the culled feature wavelength set.

Acknowledging the potential for overfitting due to the higher number of features than samples, we used the PCA method to derive principal component features from the 94 feature bands initially screened with SCARS, resulting in the extraction of 5 principal components. Termed the SCARS-PCA features, these five components exhibit contribution rates of 83.26%, 7.88%, 6%, 1.57%, and 0.63% respectively. Notably, their cumulative contribution rate stands at 99.34%, signifying their robust retention of original sample information.

#### 2.2.3. Regression Algorithm

In the realm of predictive modeling for soil organic matter content, the prevailing linear regression techniques encompass PLSR and LR. Conversely, non-linear regression methodologies encompass SVM and RF. The linear regression algorithm endeavors to locate an optimal regression line that minimizes the collective deviation of the sample dataset, thus determining the optimal regression coefficients. Ridge regression, serving as a bias-correction estimation method for covariate data analysis, modifies the least squares estimation approach. This adaptation prevents overfitting by introducing a constraint term, representing a singular parameter, into the cost function. Furthermore, the Ridge Cross-Validation (RidgeCV or RCV) method, equipped with an intrinsic parameter, mirrors lattice search procedures. It integrates cross-validation to appraise the model, conducts an automatic search within a predefined range, and subsequently determines the optimal coefficients for the constraint terms. SVM, a potent algorithm, orchestrates classification or prediction tasks through the identification of hyperplanes for sample segregation. If samples prove intractable to linear partitioning within their native dimensional space, the kernel function comes into play, facilitating projection into a higher-dimensional feature space. RF stands as an amalgamated learning approach that operates on the foundational concept of assembling numerous decision trees distinguished by varying parameters. In this method, each decision tree undertakes individual predictions, and ultimately, the collective prediction outcome emerges as an average of all decision tree prognoses. Extra Randomized Trees (ExtraTrees), akin to RF, embrace a kindred methodology. The demarcation between the two methodologies resides in their respective implementation nuances. RF uses the Bagging technique, entailing the random selection of samples to train each decision tree. This selection process aims to derive optimal branching attributes within a randomized subset. In contrast, ExtraTrees uses the entirety of the samples for training each decision tree, embracing a distinct approach where the optimal branching attributes are selected in a fully arbitrary manner. In essence, ExtraTrees not only utilizes the same samples for all decision trees but also assigns its optimal bifurcation attributes using a completely random selection process. Bagging and boosting are techniques that amalgamate weak classifiers to forge robust classifiers, effectively heightening model efficacy through the synergy of multiple models. Within this framework, the Gradient Boosting Decision Tree (GBDT) surfaces as a decision tree model propelled by the unification concept with the application of the boosting methodology. The trio of CatBoost, XGBoost, and LightGBM represent the foremost constituents of the GBDT arsenal, all constituting enhanced iterations within the broader GBDT algorithm framework. In contrast to XGBoost and LightGBM, CatBoost introduces an innovative algorithmic approach that seamlessly converts categorical features into numerical counterparts. It leverages feature interconnections to amalgamate categorical attributes, resulting in a substantial augmentation of feature dimensionality. Moreover, CatBoost uses a sort boosting mechanism that adeptly mitigates noise interference during training. By circumventing the gradient estimation bias, it effectively addresses prediction biases [24].

To assess the efficacy of the feature fusion methodology in enhancing model prediction performance, this study uses the amalgamated features as independent variables and the normalized soil organic matter content as the dependent variable. Various regression methods are chosen as estimators to establish the linkage between the fused features and soil organic matter content. The hyperparameter configurations for these regression algorithms are presented in Table 3, with unlisted parameters adhering to default values. As an illustrative example, within the XGBoost algorithm, the parameter n_estimators signifies the number of iterations, learning_rate represents the learning rate, max_depth determines the maximum tree depth, subsample denotes the proportion of subsamples utilized in training, colsample_bytree signifies the proportion of randomly selected features during tree construction, and tree_method designates the tree constraint algorithm.

#### 2.2.4. Metrics for Model Assessment

In this study, the coefficient of determination (R^2^) and Root Mean Square Error (RMSE) serve as the pivotal benchmarks for evaluating the prediction models, as outlined in Equations (3) and (4), respectively. Here, *n* represents the sample count, ${\hat{y}}_{i}$ and $y_{i}$ signify the predicted and actual values of the *i*th sample, correspondingly, and $\bar{y}$ denotes the mean of the actual values. Notably, a larger R^2^ and a smaller RMSE indicate heightened model prediction precision, reflecting a closer proximity of the predicted values to the true values.

(3) $$R^{2}=1-\frac{\sum_{i=1}^{n}{\left({\hat{y}}_{i}-y_{i}\right)}^{2}}{\sum_{i=1}^{n}{\left(y_{i}-\bar{y}\right)}^{2}}$$

(4) $$RMSE=\sqrt{\frac{\sum_{i=1}^{n}{\left({\hat{y}}_{i}-y_{i}\right)}^{2}}{n}}$$

## 3. Results

Data processing, model construction, and evaluation were executed on the PyCharm Community Edition 2019.3.1 ×64 platform, operating within a Windows environment. During data preprocessing, the procedures encompassed encoding and normalization of soil profile data. Additionally, feature engineering was accomplished by conducting PCA feature extraction, Lasso, and SCARS feature wavelength selection for spectral attributes. In the realm of SOM content prediction using PCA features of full-band spectra, diverse strata of soil profile attributes, Lasso feature wavelengths, SCARS-PCA features, and amalgamated features, the sample dataset was methodically partitioned using the train_test_split function. This process allocated 80% of the dataset for model training while reserving the remaining 20% for uninvolved model assessment and validation. Notably, pivotal parameters such as test_size = 0.2 and random_state = 0 were configured in this partitioning endeavor. To evaluate the efficacy of this fusion feature strategy, the models were subjected to rigorous assessment utilizing indicators such as R^2^ and RMSE. Comparative analyses were conducted among models built upon PCA features of full-band spectral data, soil profile features, Lasso-selected features, SCARS-PCA features, and the proposed fusion features. Each model underwent validation on a test dataset, with performance metrics meticulously documented.

### 3.1. Prediction of SOM Content Using Single-Type Features

To comprehensively assess the efficacy of the SOM content prediction model under distinct feature contexts and, concurrently, establish a baseline for the proposed fusion feature model, this study undertook predictive modeling of SOM content. Specifically, modeling was conducted using PCA features of full-band spectra, soil profile attributes, Lasso-selected features, and SCARS-PCA features. The outcomes of model evaluations are meticulously presented in Table 4 and Table 5 for thorough analysis.

In the context of the PCA features of the full-band spectra modeling, the independent variable comprises five PCA features, while the dependent variable is the normalized SOM content. With the integration of regression algorithms, a predictive model for SOM content was constructed. The performance evaluation metrics for each model are showcased in the leftmost four columns of Table 4. A comparative analysis of the assessment metrics from PCA features modeling reveals that non-linear regression techniques (such as RF, SVM, CatBoost, LightGBM, ExtraTrees, and XGBoost) exhibit a great advantage over linear regression methods (RCV, LR, and PLSR) on the training dataset. The performance of these regression models on the test set is notable, with XGBoost leading the pack (R^2^ and RMSE of 0.798 and 0.115, respectively), closely followed by SVM, ExtraTrees, and RF. Given the considerable number of bands in the full-band spectra, the presence of redundant bands could potentially disrupt SOM content predictions, resulting in deviations in model fitting outcomes. The regression algorithm’s performance on the test set indicates that using PCA for full-band spectral feature extraction alleviates the influence of redundant bands, enhancing the predictive capacity of the regression model.

In the domain of profile feature modeling, the 12 pre-processed profile features were used as independent variables, while normalized SOM content served as the dependent variable. By harnessing the synergy of regression algorithms, a model for estimating SOM content was forged. The evaluation metrics pertaining to model performance are presented in the rightmost four columns of Table 4. Upon scrutinizing the outcomes in Table 4, it is evident that the R^2^ metrics of LR, PLSR, and SVM on the test dataset are notably low, indicative of their subpar fitting performance. Conversely, RF, CatBoost, ExtraTrees, and XGBoost—comprising integrated learning methodologies—exhibit superior performance. These models showcase R^2^ metrics exceeding 0.73. In summation, the regression models constructed around diverse hierarchical profile features effectively facilitate the inversion of SOM content. Nevertheless, analogous to the PCA features of full-band spectra modeling, the model’s fitting efficacy is deemed merely satisfactory, leaving ample scope for enhancing prediction performance.

The R^2^ and RMSE metrics were obtained by crafting the SOM content prediction model with 11 feature wavelengths meticulously screened with the Lasso method as independent variables and normalized SOM content as the dependent variable. Their values are meticulously presented in the leftmost four columns of Table 5. In contrast with the findings of the comprehensive PCA features of full-band spectra modeling, the SOM content prediction model, engineered upon the foundation of Lasso-selected features, underscores a noticeable uplift in the R^2^ metrics for RCV and LightGBM on the training set. Meanwhile, the variances across the other models remain rather inconspicuous. Upon scrutinizing the outcomes on the test dataset, disparities in the R^2^ values are minimal, with variations below 0.02 for RCV, LR, and PLSR. Conversely, the R^2^ values of RF, SVM, LightGBM, ExtraTrees, and XGBoost display a remarkable decrease. Of noteworthy significance is the remarkable enhancement witnessed in CatBoost, where its efficacy escalates from 0.707 to 0.762. The R^2^ values obtained from the SOM content prediction model, leveraging features selected with Lasso, affirm that integrating these attributes into SOM content prediction yields less enhancement in the regression model’s performance compared with modeling with PCA features. This discrepancy might arise from the omission of certain bands containing crucial information during the characteristic band screening using the LASSO method.

Utilizing five PCA features of the meticulously screened 94 feature wavelengths, which were selected with the SCARS feature selection method, as independent variables and normalized SOM content as the dependent variable, a regression model tailored to SCARS-PCA features was formulated. The outcome of this endeavor is vividly portrayed in the rightmost four columns of Table 5. Upon meticulous examination of this tabulated data, it becomes evident that the R^2^ and RMSE metrics, characterizing the performance of the SCARS-PCA features-centric SOM content prediction model on the test dataset, are confined to the intervals [0.627, 0.837] and [0.103, 0.157], respectively. Comparing these metrics against the backdrop of the comprehensive PCA features of full-band spectra modeling, a few compelling trends emerge. The R^2^ metrics demonstrate notable advancements in the RCV, LR, PLSR, and ExtraTrees models, effectively ameliorating their predictive prowess, while a marginal decrease is discernible for the RF, SVM, CatBoost, LightGBM, and XGBoost models. Within the SOM content prediction model constructed using SCARS-PCA features, several regression methods exhibit superior R^2^ metrics compared with the LASSO-screened feature modeling. Notably, RCV, LR, PLSR, RF, LightGBM, and ExtraTrees stand out, with ExtraTrees demonstrating the most remarkable performance. This method achieved the highest R^2^ metric of 0.837 and the lowest RMSE value of 0.103 for a singular SOM content feature prediction on the test set.

Upon a thorough juxtaposition of the R^2^ metrics of the regression models elucidated in both Table 4 and Table 5, it becomes manifestly clear that none of the SOM content prediction models formulated on the grounds of singular feature types exhibit a pronounced edge in performance. In a holistic appraisal, it emerges that the capacity of individual feature categories to explicate soil organic matter content remains circumscribed. Evidently, ample scope for enhancing the predictive efficacy of regression models persists.

### 3.2. Prediction of SOM Content Using Fusion Features

The fusion of the five PCA principal component features from full-band spectra with the 12 profile features served as independent variables, while the normalized SOM content was utilized as the dependent variable. A combination of regression algorithms was used to construct prediction models for SOM content. The evaluation metrics of these models are presented in the first four columns of Table 6. Notably, on the training set, the RCV, LR, and PLSR linear regression models showcased substantial improvements in the R^2^ metrics compared with the models based solely on PCA features from full-band spectra or profile features. On the test set, RCV, LR, PLSR, SVM, and CatBoost demonstrated commendable performance, exhibiting R^2^ metric values consistent with those of single-type feature modeling. However, RF, LightGBM, ExtraTrees, and XGBoost exhibited notable enhancements in R^2^ metric values, particularly ExtraTrees, which displayed exceptional performance with an R^2^ value as high as 0.931 (corresponding to an RMSE value of 0.068). This represents a significant enhancement rate of 17% and 26%, respectively, followed closely by RF and XGBoost, both surpassing an R^2^ value of 0.87. The comparative analysis underscores that the fusion of spectral principal component features with complementary profile features has a substantial positive impact on the predictive performance of RF, ExtraTrees, and XGBoost models for SOM content prediction.

By harmonizing the strengths of the Lasso-selected features with intricate soil profile features, a comprehensive ensemble of 23 features was cultivated. Leveraging these fused features, this study aimed to construct a robust model for predicting SOM content. The outcomes of this integrated model, coupled with a suite of regression algorithms, were meticulously evaluated using a comprehensive set of metrics. The findings, succinctly encapsulated in the central four columns of Table 6, highlight the effectiveness of the fusion feature methodology. Remarkably, the regression algorithms’ performance metrics on the test dataset exhibited substantial improvements, with R^2^ and RMSE values within the [0.687, 0.907] and [0.078, 0.144] ranges, respectively. This indicates high precision in predicting SOM content, particularly within the context of ExtraTrees, which emerged as the standout performer, boasting R^2^ and RMSE values of 0.907 and 0.078, respectively. Comparative analyses against models reliant on single-feature types, such as soil profile features and Lasso-selected features, emphasized the superiority of the fusion feature strategy. Notably, the fusion of Lasso-selected features and profile features exhibited superior fitting goodness. Noteworthy performance enhancements were observed across the spectrum of regression methods, especially within the ambit of integrated learning methods. For instance, the R^2^ metrics on the test set showcased substantial gains: RF witnessed an ascent from 0.746 and 0.718 (for single-type feature modeling) to an impressive 0.885 (with growth rates of 19% and 23%, respectively). Similarly, CatBoost exhibited an elevation from 0.732 and 0.762 to a noteworthy 0.883 (demonstrating growth rates of 21% and 16%, respectively). ExtraTrees registered a remarkable improvement, surging from 0.738 and 0.738 to an exceptional 0.907 (signifying growth rates of 23% and 23%). Moreover, XGBoost experienced a remarkable surge, escalating from 0.767 and 0.759 to a notable 0.892 (reflecting growth rates of 26% and 18%, respectively). Evidently, the fusion of Lasso-selected features with profile features, integrated within the framework of RF, CatBoost, ExtraTrees, and XGBoost models, holds substantial promise in significantly elevating the predictive prowess of the model. This innovative approach stands poised to revolutionize the prediction of SOM content and underscores its pertinence in soil science research.

The synergy of five PCA features of 94 SCARS-derived wavelengths with intricate profile features yielded a comprehensive ensemble of 17 features. Leveraging this fusion feature strategy, the focus was on building a robust regression model for predicting SOM content. The outcomes of this innovative approach, coupled with diverse regression algorithms, were meticulously assessed using a comprehensive suite of evaluation metrics. The results, elegantly presented in the four rightmost columns of Table 6, underscore the superior efficacy of the SCARS feature fusion technique. Notably, the R^2^ and RMSE metrics of the SCARS-PCA features-fused profile features modeling on the test dataset exhibited impressive values within the [0.711, 0.915] and [0.075, 0.138] range, respectively. Among the standout performers, RF took the lead, displaying remarkable R^2^ and RMSE metrics of 0.915 and 0.075, respectively, closely followed by ExtraTrees and XGBoost. A closer examination of the metrics reveals the exceptional capabilities of the RF model based on fusion features. With an impressive enhancement of 23% and 24% in R^2^ metrics compared with profile features and SCARS-PCA features modeling, respectively, the RF model stands as a testament to the potential of this integration strategy. ExtraTrees exhibited notable predictive prowess, elevating the R^2^ metric value from 0.738 in profile feature modeling and 0.837 in SCARS-PCA feature modeling to an impressive 0.903. Simultaneously, the RMSE value decreased from 0.131 and 0.103 to 0.080. Collectively, these findings validate the supremacy of the SCARS-PCA features-fused profile features, synergized with the RF and ExtraTrees algorithms, in attaining unparalleled prediction accuracy. This cutting-edge approach has the potential to revolutionize SOM content prediction, marking a significant advancement in the realm of soil science research.

Among the various feature fusion strategies, RF, ExtraTrees, and XGBoost exhibit exceptional predictive performance on the test set. Not only do they surpass their counterparts, but they also demonstrate high prediction accuracy, solidifying their status as premier choices among regression models for SOM content prediction. This heightened performance could be attributed to the fusion features we engineered and the operational mechanisms of these models. All three algorithms represent integrated models, leveraging the aggregation of multiple models’ predictions to offer more dependable results, thereby mitigating the bias and variance inherent in individual models. RF and ExtraTrees, both utilizing multiple decision trees, introduce randomness during training. While RF utilizes a bootstrap method with randomized put-back sampling for training each decision tree, ExtraTrees differs by utilizing all training samples but with random feature selection for each tree. The fusion strategy constructed features that integrated spectral features with environmental soil attributes, leveraging complementary advantages between these features. Introducing randomness in RF and ExtraTrees renders them less susceptible to overfitting, enhancing noise immunity when constructing the soil organic matter prediction model using these fused features. XGBoost, an enhanced version of GBDT, exhibits remarkable performance within the soil organic matter prediction model constructed with the fusion strategies. Its efficacy may stem from second-order Taylor expansion of the loss function, supplementing the objective function with a regular term, and a comprehensive search for the optimal solution. This approach effectively mitigates overfitting, contributing to its superior performance.

## 4. Discussion

### 4.1. Lasso-Selected Features’ Impact on SOM Prediction Models

**Figure 5:**
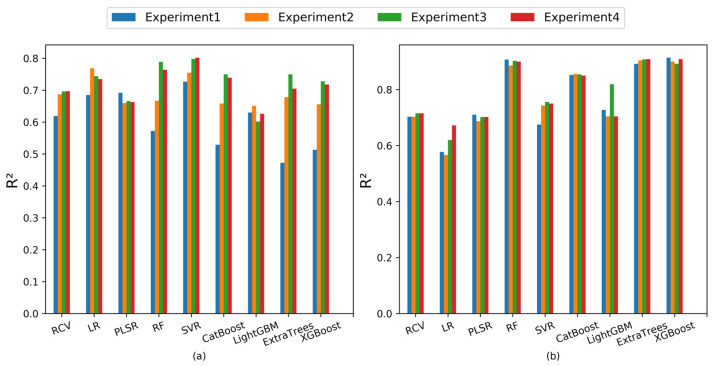
The *R*^2^ values of the regression models on the test set in the replication experiments. (**a**) Lasso-selected features modeling; (**b**) Lasso-selected features-fused profile features modeling.

In Lasso feature selection, a defined range of alphas is used, determined with cross-validation to sieve the optimal values. These values are pivotal in screening the feature bands from the full-band spectrum. To investigate the impact of Lasso-screened feature bands on SOM content prediction models, we established four distinct non-overlapping alpha acquisition ranges, and the resulting screened feature bands are presented in Table 7. The parameter alphas were determined using the np.range function as np.range(0.01, 1, 0.01). In this instance, the first parameter value of 0.01 represents the starting point for the alphas, the second parameter value of 1 denotes the endpoint (exclusive), and the third parameter value of 0.01 defines the step length. Notably, the optimal alphas from these ranges varied, yielding divergent sets of feature bands. For instance, when the optimal alphas were 0.0014 and 0.00061, the resulting feature bands closely aligned, differing only by two bands, yet they substantially deviated from bands derived using other optimal alphas. Utilizing these filtered feature bands and their combinations with profile features, we constructed prediction models, evaluating their R^2^ indices on the test set (Figure 5) and corresponding statistical results (Table 8).

Among the four Lasso screening feature models, SVM displayed superior performance, boasting an average R^2^ value and standard variance of 0.771 and 0.036, respectively. LR closely followed with average R^2^ value and standard variance of 0.733 and 0.035, respectively. However, the remaining models exhibited more general performance, with average R^2^ values not surpassing 0.7. Particularly, ExtraTrees showcased the largest standard deviation, notably influenced by the Lasso feature band. Among the four sets of fused-feature modeling, RCV, PLSR, RF, CatBoost, ExtraTrees, and XGBoost consistently exhibited stable performances, with none surpassing a standard deviation of 0.01. Noteworthy among them, RF, ExtraTrees, and XGBoost consistently presented higher mean R^2^ values and smaller standard deviations. They adeptly fit regression model parameters from fused features, demonstrating superior performance on the test set. Comparing different Lasso screening feature fusion models, RF, ExtraTrees, and XGBoost models for soil organic matter prediction, exhibited enhanced stability and superior fitting.

### 4.2. SCARS-Selected Features’ Impact on SOM Prediction Models

**Figure 6:**
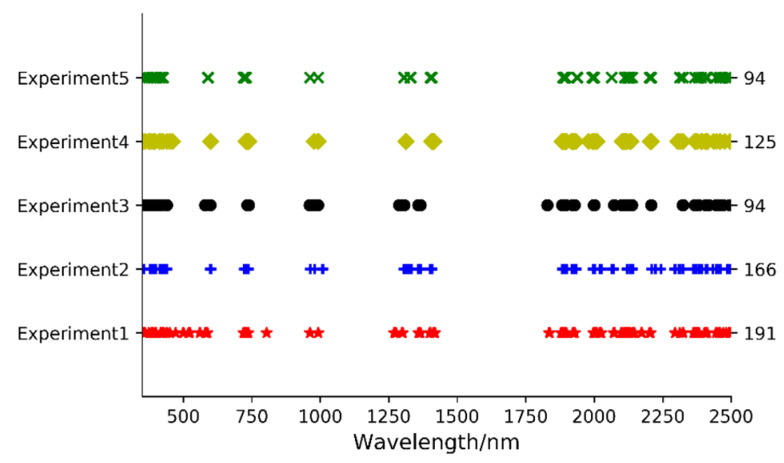
Distribution of feature wavelengths selected with SCARS in 5 rounds of experiments.

**Figure 7:**
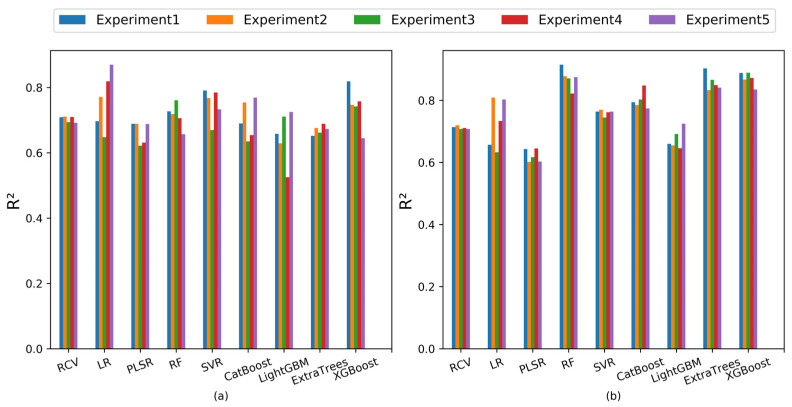
The *R*^2^ values of regression models on the test set in the replication experiments. (**a**) SCARS-PCA features modeling and (**b**) SCARS-PCA features-fused profile features modeling.

When using SCARS feature selection, the inherent stochasticity of Monte Carlo sampling introduces variability in both the count and positions of the selected feature wavelengths. To comprehend the stability and robustness of the SOM content estimation model stemming from SCARS-PCA features, we conducted five rounds of the feature wavelength selection and PCA experiment. The resultant distribution of feature wavelengths picked across these trials is visually depicted in Figure 6. This analysis illustrates that while the selected wavelengths differ in location between experiments, a notable coherence exists in the overall range of wavelength distributions. Intriguing insights emerge from the R^2^ indices of the SOM content prediction model based on the SCARS-PCA features and their fusion with profile features on the test set, as depicted in Figure 7. Accompanied by the in-depth statistical analysis presented in Table 9, these results unveil a nuanced picture of model stability and performance. Figure 7a and Table 9 underscore that when modeling relies solely on SCARS-PCA characteristics, notable fluctuations in the R^2^ indices of several models emerge. This variance suggests that the stability of these models is limited, with only RCV showcasing better stability. However, a different trend emerges when analyzing Figure 7b in conjunction with Table 9. For fusion feature modeling, with SCARS-PCA features integrated, a more stable performance prevails across most models, apart from LR, RF, and LightGBM, which display relatively weaker stability (as indicated by a standard deviation of R^2^ exceeding 0.03). A comprehensive assessment of prediction accuracy and stability underscores that the SCARS-PCA features fusion model demonstrates comparable stability to the SCARS-PCA features modeling itself. Impressively, the RF, ExtraTrees, and XGBoost model, propelled by SCARS-PCA features harmonized with profile features, emerges as the pinnacle performer.

## 5. Conclusions

This paper introduces an innovative approach to predict SOM content by merging distinct feature sets: PCA features, Lasso-selected features, and SCARS-PCA features, alongside soil profile features from sampling points. This integration aims to bolster prediction accuracy and stability, addressing the intricate dynamics of SOM. The model’s efficacy is verified using soil profile data, spectral information from soil samples, and measured SOM content collected from rice field sites in and around Changsha.

The experimental results unveil a remarkable improvement in predictive performance compared with models relying solely on PCA features from full-band spectra, Lasso-selected features, and SCARS-PCA features for SOM content prediction. The proposed fusion-based SOM prediction model, particularly leveraged by regression algorithms like RF, ExtraTrees, and XGBoost, emerges as a frontrunner in terms of marked enhancements in goodness-of-fit. Among these algorithms, RF and ExtraTrees exhibit substantial advancements, showcasing superior performance. This study delves into the variability intrinsic to Lasso-selected features and SCARS-PCA features, emphasizing the stability achieved in the resulting model. With comprehensive experimentation, it is evident that the fusion model built on Lasso-selected features and SCARS-PCA features consistently attains higher prediction accuracy and stability, especially when coupled with the RF, ExtraTrees, and XGBoost algorithms.

In summary, this paper introduces a pioneering fusion-driven model for predicting SOM content, seamlessly amalgamating spectral feature wavelengths with soil profile characteristics. By effectively addressing the limitations of individual feature sets, this approach signals a paradigm shift toward augmented prediction accuracy and resilience. The findings underscore the efficacy of regression algorithms like RF, ExtraTrees, and XGBoost in harnessing fusion features’ potential to optimize SOM content predictions. The comprehensive methodology outlined in this study makes a significant contribution to the realms of soil science and predictive modeling.

## Figures and Tables

**Table 1:** Profile information of CS-03.

Feature Name	CS-03-Aa	CS-03-Ap	CS-03-B	CS-03-Br	CS-03-Bg	CS-03-Er
Profile_level	1	2	3	4	5	6
Color_class	7.5	5.5	10	10	10	2.5
Color_value	4	4	4	3	2	5
Color_chroma	4	6	4	3	2	3
Plant_root_thickness	Medium	Thin	Thin	Minuteness	None	None
Plant_root_abundance	Many	few	few	Seldom	None	None
Degree_of_soil_ structure_development	Strong	Strong	Medium	Weak	Weak	Weak
Porosity	High	Medium	Medium	Medium	Medium	Medium
Pore_size	Medium	Thin	Thin	Minuteness	Minuteness	Minuteness
Pore_abundance	Medium	Few	Few	Seldom	Seldom	Seldom
Plasticity	Medium	Medium	Medium	Medium	Strong	Strong
pH	9	8.5	8.5	8.5	8.2	8.2

**Table 2:** The outcomes following the data preprocessing of CS03.

Feature Name	CS-03-Aa	CS-03-Ap	CS-03-B	CS-03-Br	CS-03-Bg	CS-03-Er
Profile_level	0.947	0.578	0.315	0.234	0.142	0.066
Color_class	0.287	0.152	0.021	0.019	0.049	0.324
Color_value	0.675	0.676	0.684	0.896	0.466	0.218
Color_chroma	0.761	0.376	0.779	0.892	0.824	0.988
Plant_root_thickness	0.676	0.403	0.412	0.101	0.015	0.020
Plant_root_abundance	0.885	0.416	0.431	0.045	0.018	0.024
Degree_of_soil_ structure_development	0.924	0.927	0.707	0.237	0.294	0.311
Porosity	0.855	0.345	0.356	0.354	0.386	0.396
Pore_size	0.838	0.739	0.754	0.325	0.389	0.407
Pore_abundance	0.220	0.008	0.017	0.031	0.079	0.093
Plasticity	0.930	0.934	0.945	0.943	0.633	0.654
pH	0.292	0.775	0.872	0.856	0.000	0.080

**Table 3:** Hyperparameter configuration for algorithms.

Algorithm	Hyperparameter Configuration
RCV	alphas = np.arange (1, 10, 0.2)
LR	Normalize = False
PLSR	Normalize = False
RF	n_estimatiors = 300, criterion = ‘mse’, max_depth = 7
SVM	Kernel = ‘rbf’
CatBoost	Iterations = 100, depth = 10
LightGBM	Objective = ‘regression’, n_estimations = 300
ExtraTrees	Criterion = ‘mse’, min_samples_split = 2
XGBoost	n_estimators = 300, learning_rate = 0.08, gamma = 0, subsample = 0.75, colsample_bytree = 1, max_depth = 7, tree_method = ‘approx’

**Table 4:** Performance evaluation of PCA features of full-band spectra and profile features modeling.

Algorithm	Five PCA Features of Full-Band Spectra				Twelve Profile Features			
	Training Set		Test Set		Training Set		Test Set	
	R^2^	RMSE	R^2^	RMSE	R^2^	RMSE	R^2^	RMSE
RCV	0.684	0.172	0.680	0.145	0.709	0.165	0.633	0.155
LR	0.688	0.170	0.661	0.149	0.740	0.156	0.484	0.184
PLSR	0.688	0.170	0.661	0.149	0.711	0.164	0.596	0.163
RF	0.950	0.068	0.780	0.120	0.954	0.065	0.746	0.129
SVM	0.810	0.133	0.797	0.116	0.876	0.107	0.561	0.170
CatBoost	0.970	0.053	0.707	0.139	0.978	0.022	0.732	0.133
LightGBM	0.830	0.126	0.687	0.144	0.945	0.071	0.618	0.159
ExtraTrees	0.974	0.049	0.795	0.119	0.986	0.020	0.738	0.131
XGBoost	0.978	0.020	0.798	0.115	0.961	0.031	0.767	0.124

**Table 5:** Performance evaluation of Lasso-selected features and SCARS-PCA features modeling.

Algorithm	Eleven Lasso-Selected Features				Five SCARS-PCA Features			
	Training Set		Test SET		Training Set		Test Set	
	R^2^	RMSE	R^2^	RMSE	R^2^	RMSE	R^2^	RMSE
RCV	0.732	0.161	0.684	0.144	0.756	0.151	0.759	0.126
LR	0.670	0.175	0.660	0.150	0.802	0.136	0.695	0.142
PLSR	0.679	0.173	0.646	0.153	0.802	0.136	0.695	0.142
RF	0.946	0.071	0.718	0.136	0.958	0.062	0.738	0.131
SVM	0.760	0.149	0.757	0.127	0.851	0.118	0.729	0.134
CatBoost	0.965	0.052	0.762	0.125	0.955	0.073	0.627	0.157
LightGBM	0.923	0.085	0.651	0.152	0.918	0.087	0.668	0.148
ExtraTrees	0.988	0.012	0.738	0.131	0.985	0.016	0.837	0.103
XGBoost	0.980	0.018	0.759	0.126	0.946	0.072	0.712	0.138

**Table 6:** Performance evaluation of fusion features modeling.

Algorithm	Five PCA Features of Full-Band Spectra and 12 Profile Features				Eleven Lasso-Selected Features and 12 Profile Features				Five SCARS-PCA Features and 12 Profile Features			
	Training Set		Test Set		Training Set		Test Set		Training Set		Test Set	
	R^2^	RMSE	R^2^	RMSE	R^2^	RMSE	R^2^	RMSE	R^2^	RMSE	R^2^	RMSE
RCV	0.850	0.118	0.694	0.142	0.814	0.132	0.706	0.139	0.864	0.112	0.718	0.136
LR	0.868	0.111	0.609	0.160	0.858	0.115	0.769	0.123	0.957	0.063	0.749	0.128
PLSR	0.842	0.121	0.634	0.155	0.829	0.126	0.687	0.144	0.805	0.135	0.711	0.138
RF	0.976	0.048	0.896	0.083	0.974	0.049	0.885	0.087	0.975	0.048	0.915	0.075
SVM	0.912	0.091	0.785	0.119	0.916	0.088	0.815	0.110	0.921	0.086	0.774	0.122
CatBoost	0.964	0.050	0.695	0.142	0.963	0.052	0.883	0.095	0.965	0.058	0.813	0.111
LightGBM	0.971	0.052	0.808	0.113	0.948	0.069	0.760	0.126	0.955	0.064	0.723	0.135
ExtraTrees	0.964	0.053	0.931	0.068	0.968	0.051	0.907	0.078	0.950	0.064	0.903	0.080
XGBoost	0.948	0.069	0.874	0.091	0.941	0.071	0.892	0.084	0.953	0.061	0.888	0.086

**Table 7:** Parameter configuration for Lasso feature selection and results in the replication experiments.

Experiment	Alpha Configuration	Optimal Alpha	Selected Wavelength
1	np.arange (0.01, 1, 0.01)	0.01	[‘770’, ‘772’, ‘773’, ‘774’, ‘775’, ‘1279’]
2	np.arange (0.001, 0.01, 0.0001)	0.0014	[‘353’, ‘767’, ‘768’, ‘769’, ‘770’, ‘1356’, ‘1357’, ‘1358’, ‘1359’]
3	np.arange (0.0001, 0.001, 0.00001)	0.00061	[‘353’, ‘767’, ‘768’, ‘769’, ‘770’, ‘1356’, ‘1357’, ‘1358’, ‘1359’,‘1360’, ‘2486’]
4	np.arange (0.00001, 0.0001, 0.000001)	0.000099	[‘353’, ‘610’, ‘611’, ‘612’, ‘613’, ‘614’, ‘1355’, ‘1356’, ‘1357’,‘1358’, ‘1359’, ‘1360’, ‘2408’]

**Table 8:** Descriptive statistics of *R^2^* values of Lasso-selected features modeling in the replication experiments.

Algorithm	Lasso-Selected Features Modeling				Lasso-Selected Features and 12 Profile Features			
	Mean	Standard Deviation	Minimum	Maximum	Mean	Standard Deviation	Minimum	Maximum
RCV	0.675	0.037	0.619	0.697	0.709	0.007	0.703	0.715
LR	0.733	0.035	0.685	0.769	0.608	0.048	0.566	0.672
PLSR	0.670	0.014	0.660	0.692	0.700	0.010	0.687	0.710
RF	0.698	0.099	0.572	0.789	0.899	0.009	0.886	0.907
SVM	0.771	0.036	0.727	0.802	0.731	0.038	0.675	0.756
CatBoost	0.669	0.102	0.529	0.750	0.853	0.003	0.850	0.856
LightGBM	0.627	0.020	0.602	0.651	0.739	0.055	0.704	0.819
ExtraTrees	0.651	0.123	0.472	0.750	0.903	0.008	0.892	0.909
XGBoost	0.653	0.099	0.513	0.728	0.904	0.010	0.892	0.914

**Table 9:** Descriptive statistics of *R*^2^ values of SCARS-PCA features modeling in the replication experiments.

Algorithm	Five SCARS-PCA Features Modeling				Five SCARS-PCA Features and 12 Profile Features			
	Mean	Standard Deviation	Minimum	Maximum	Mean	Standard Deviation	Minimum	Maximum
RCV	0.702	0.007	0.692	0.710	0.712	0.005	0.708	0.721
LR	0.761	0.089	0.648	0.870	0.727	0.080	0.633	0.809
PLSR	0.664	0.034	0.622	0.689	0.622	0.021	0.602	0.645
RF	0.714	0.037	0.657	0.761	0.872	0.033	0.822	0.915
SVM	0.749	0.049	0.670	0.791	0.761	0.009	0.745	0.770
CatBoost	0.700	0.059	0.635	0.769	0.801	0.028	0.774	0.848
LightGBM	0.649	0.079	0.525	0.725	0.675	0.033	0.646	0.725
ExtraTrees	0.670	0.014	0.652	0.689	0.858	0.028	0.833	0.903
XGBoost	0.742	0.063	0.645	0.820	0.870	0.022	0.835	0.889

## Data Availability

Data underlying the results presented in this paper are not publicly available at this time but may be obtained from the authors upon reasonable request.

## References

[1] Zhang X.Y., Yao Y.M., Yan X.Z. (2021). Research progress on prediction of soil organic matter content by mid-infrared spectroscopy. Soil Fertil. Sci. China.

[2] Tao Z.P., Xu Z.H., Ding J.N., Zhang Y. (2022). Determination of soil organic matter content under forest based on different methods. Sci. Technol. Eng..

[3] Yumiti M.M., Wang X.M. (2022). Hyperspectral estimation of soil organic matter content based on continuous wavelet transformation. Spectrosc. Spectr. Anal..

[4] Li X., Fan Z.Q., Gao H., Zhang X.Y., Dong Y.S.P., Hong P.Z., Wang K., Liu P.Z., Du C.W., Li X.J. (2021). Construction of soil organic matter rapid detection model based on hyperspectral. J. Shandong Agric. Univ..

[5] Allo M., Todoroff P., Jameux M., Stern M., Paulin L., Albrecht A. (2020). Prediction of tropical volcanic soil organic carbon stocks by visible-near- and mid-infrared spectroscopy. Catena.

[6] Zhou W., Xie L.J., Yang H., Hua L., Li H.R., Yang M. (2021). Hyperspectral inversion of soil organic matter content in the three-rivers source region. Chin. J. Soil Sci..

[7] Shang T.H., Mao H.X., Zhang J.H., Chen R.H., Wang F., Jia K.L. (2021). Hyperspectral estimation of soil organic matter content in Yinchuan plain, China based on PCA sensitive band screening and SVM modeling. Chin. J. Ecol..

[8] Gou Y., Zhao Y., Li Y., Zhuo Z., Cao M., Huang Y. (2022). Soil organic matter content in dryland farmland in northeast China with hyperspectral reflectance based on CWT-SCARS. Trans. Chin. Soc. Agric. Mach..

[9] Liu J., Dong Z., Xia J., Wang H., Meng T., Zhang R., Han J., Wang N., Xie J. (2019). Estimation of soil organic matter content based on characteristic variable selection and regression methods. Acta Opt. Sin..

[10] Li X.Y., Fang P.P., Liu Y., Qian W., Lu M. (2019). Extracting characteristic wavelength of soil nutrients based on multi-classifier fusion. Spectrosc. Spectr. Anal..

[11] Yu L., Hong Y., Zhou Y., Zhu Q., Xu L., Li J., Nie Y. (2016). Wavelength variable selection methods for estimation of soil organic matter content using hyperspectral technique. Trans. Chin. Soc. Agric. Eng..

[12] Hao X.X. (2017). Change Characteristic of Soil Organic Matter in Mollisol Profile under Different Ecosystem. Ph.D. Thesis.

[13] Zhang X., Li M.J., Liu X.B., Wu W. (2020). Distribution characteristics and influence factors of organic matter content in cultivated soil in different horizons in hilly areas. Resour. Environ. Yangtze Basin.

[14] Gao L., Chen X., Lin C., Wang W., Zhang Y. (2018). Characteristic of soil profile and nutrient change of fragrant taro typical region in Shaoguan. Southwest China J. Agric. Sci..

[15] Jia Q.W., Liu X.F., Xiao P.Y. (2015). Composition and distribution characteristics of organic matter in soil profiles of Yancheng flats. Wetl. Sci..

[16] Xu X.B., Lu J.S., Wu Q.Y., Qing Y., Xu Z., Cao J. (2018). Prediction of soil organic matter based PCA-MLR and PCA-BPN algorithm using field VNIR spectroscopy in coastal soils of southern Laizhou bay. Spectrosc. Spectr. Anal..

[17] Yan X.Z., Yao Y.M., Zhang X.Y. (2019). The progress and prospect of soil organic matter mapping based on remote sensing technology. China Agric. Inform..

[18] Ai T.H. (2021). Some thoughts on deep learning enabling cartography. Acta Geod. Cartogr. Sin..

[19] Li Y., Liu X.L., Peng J., Li X., Wu J.L. (2018). Inversion of desert soil organic matter content using visible-infrared spectrum in southern Xinjiang. Chin. J. Soil Sci..

[20] Zhang D.H., Zhao Y.J., Qin K., Pei C.K., Zhao N.B. (2018). A review of hyperspectral multivariate information extraction models for soils. Soil Fertil. Sci. China.

[21] Zhang Z.T., Lao C.C., Wang H.F., Arnon K., Chen J.Y., Li Y. (2020). Estimation of desert soil organic matter through hyperspectral based on fractional-order derivatives and SVMDA-RF. Trans. Chin. Soc. Agric. Mach..

[22] Ma C.Y., Sun Y.Q., Wu Z.F., Zhang J., Niu Y., Hou Z., Chen J. (2021). Spatial prediction of topsoil organic matter of arable land by different models at the regional scale. Chin. J. Soil Sci..

[23] He S.F., Shen L.M., Xie H.X. (2021). Hyperspectral estimation model of soil organic matter content using generative adversarial networks. Spectrosc. Spectr. Anal..

[24] Prokhorenkova L., Gusev G., Vorobev A., Dorogush A.V., Gulin A. (2018). CatBoost: Unbiased boosting with categorical features. Adv. Neural Inf. Process. Syst..

